# Continental rifts losing driving forces can still complete breakup

**DOI:** 10.1038/s41598-025-19691-3

**Published:** 2025-10-23

**Authors:** Kuruvitage Chameera Chathuranga Silva, Eunseo Choi

**Affiliations:** Center for Earthquake Research and Information, The University of Memphis, Memphis, TN 38152 USA

**Keywords:** Solid Earth sciences, Geodynamics

## Abstract

The complex evolution of continental rift systems results from the intricate interplay of external driving forces and the rift system’s responses. For this reason, allowing plate kinematics to emerge from the force balance can provide deeper insights than imposing prescribed velocity boundary conditions. This study investigates the influence of temporally varying driving forces, possibly resulting from changes in slab dynamics, on rift evolution using numerical and semi-analytical models. We examined the effects of varying the timing ($t_i$), duration ($\delta t$), and magnitude ($\delta \tau$) of boundary traction reductions on extension velocities ($V_\text {E}$). Our models demonstrate that later initiation of traction reduction and slower reduction rates promote continental breakup. A 25% reduction in boundary traction can still lead to continental breakup under optimal conditions, while a 50% reduction generally results in failed rifts. Non-monotonic $V_\text {E}$ evolution, including temporary velocity increases during force reduction, is observed and explained by dynamic force balance. Our results show that a continental rift can accelerate towards breakup even when it is currently extending slowly due to a reduced driving force that can arise from many different situations.

## Introduction

Continental rifting does not invariably culminate in the complete rupture of the lithosphere and formation of new oceanic basins. Examples of such “failed” rift systems include Mid-Continent Rift in North America^1,2^, the Mississippi Embayment^3,4^, and West and Central African rift system^5,6^. The Mid-Continent Rift formed during formation of supercontinent Rodinia, which failed to split the Laurentia and reach the seafloor spreading stage^2^. Mississippi Embayment formation was related to crustal extension during breakup of the Rodinia super continent in latest Precambrian to Cambrian, during which continental extension started in the Mississippi Embayment but the focus of extension jumped eastward leaving the Mississippi Embayment as a failed rift^7^. The West and Central African rift system initiation was related to Jurassic-Early Cretaceous opening of south and central Atlantic, and continue to develop until the end of Cretaceous, where rifting was terminated due to compressional event that lead to inversions in some basins^6^. All of these examples suggest that rifts may fail in a variety of situations, but ultimately, failure occurs when driving forces become insufficient to overcome lithospheric strength.

Rift systems often display much more complex behaviors than the binary modes implied above. For instance, the Western Antarctic Rift System (WARS) has gone through multiple episodes of reactivation after a period of dormancy^8,9^. Similarly, the North Sea rift underwent multiphase extension, the first episode related to the breakup of the Pangean supercontinent^10^ and the second one linked to the deflation of central North Sea thermal dome which generated regional tensional stresses^11^. Thermo-chronological data suggest that the northern Kenya Rift experienced a rifting episode in early Cenozoic^12^, followed by a renewed extension initiated in the middle Miocene times. Furthermore, kinematic plate reconstruction modeling suggests multi-directional and multiphase pre-rift extension during South Atlantic opening^13^. These examples highlight the complex, multiphase nature of rift system evolution.

The complexity observed in rifting processes arises from the dynamic interplay between driving forces and lithospheric strength, both of which evolve non-linearly over time^14^. Driving forces, notably those exerted by subducting slabs^15^, are subject to variations in magnitude and direction. These variations can result from slab interactions with the 660 km discontinuity^16–18^, or from slab tears and detachments^19–21^, with even the duration of detachment impacting rifting^22^. Concurrently, lithospheric strength fluctuates significantly during rifting. This variability stems from changes in lithospheric thickness and thermal state, coupled with the high sensitivity of mineral creep to strain rates and temperature^23–25^.

Understanding rifting as a process of evolving balance between internal and external forces has been the focus of numerous studies. Early work generally explored lithosphere under horizontally applied stresses and its strength variations in relation to thermal gradients, crustal thickness and composition^26–29^. One-dimensional models for lithosphere going through uniform thinning have been used for understanding how strain rates evolve in such lithosphere under various assumptions on the driving force magnitudes, dry, wet or strain hardening rheology, and initial crustal and lithospheric thicknesses^30–32^. Notably, only a slight shift in the balance between driving force and strengthening by cooling was found to flip the end state of continental rifting, runaway break-up or rift failure^30^. Similar One-dimensional models were used for investigating the conditions for successful rifting in the back-arc basins along the margins of the Eurasia plate^33,34^. By examining driving force magnitudes inferred from basin subsidence, they found that the back-arc extensions were sufficiently fast for override the strengthening effects of thermal relaxation and crustal thinning. Their analysis further indicated that back-arc basin formation required a thin, hot lithosphere and a wet rheology. More recently, two-dimensional numerical models with a realistic rheology were used for showing that rift strength reduction, even with a constant driving force, is sufficient for explaining the acceleration of continental rifting prior to breakup, as observed at major continental margins^35^.

We present a novel investigation into the dynamic response of continental rifts to time-varying boundary traction, simulating the evolving magnitude of plate driving forces. We examine scenarios where driving forces, initially sufficient for continental breakup, diminish over time, which were largely unexplored in previous studies. This approach allows us to determine the conditions that lead to rift failure and to delineate the governing processes. While acknowledging the exclusion of magmatic and diking influences, known to be important^36^, our models provide a unique and insightful perspective.

## Results

This section presents the primary outcomes of our numerical simulations, detailing the processes of continental rifting and breakup under the imposed time-dependent boundary conditions. We first illustrate the overall kinematic evolution of the rift system across various reduction factors, highlighting key stages from initial deformation to continental separation or failure. Subsequently, we provide detailed analyses of the viscosity and lithospheric thickness evolution. Finally, we quantify and discuss the resulting plate boundary forces and their contribution to the observed rift dynamics within our distinct model scenarios.

### End-member reference models

**Fig. 1:**
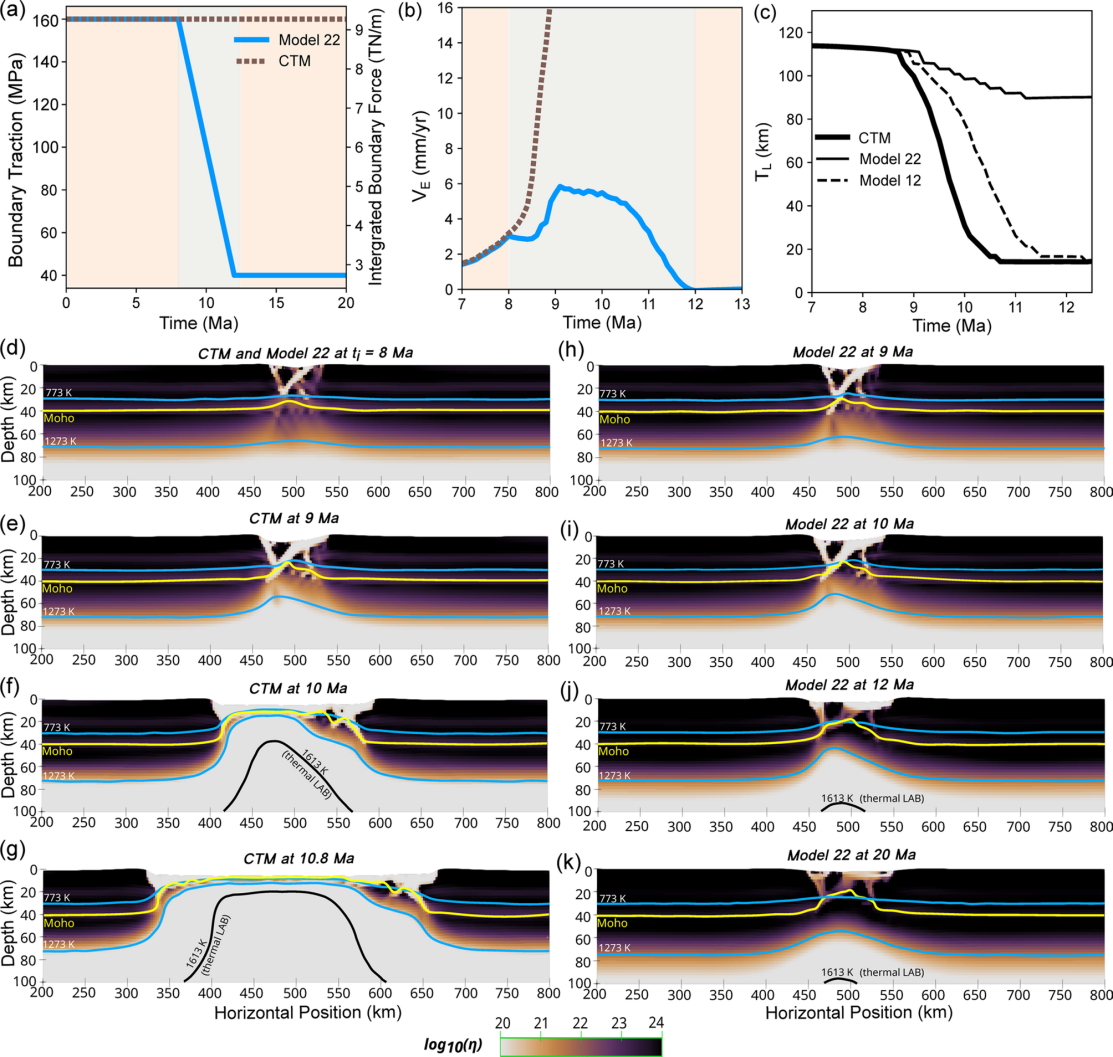
(**a**) Assigned traction magnitudes for the constant traction model (CTM) (dashed brown line) and for model 22 where 75% force reduction (Model 22) occurs over 4 Ma (solid blue line). The gray region corresponds to the period of traction magnitude reduction. (**b**) Evolution of $V_\text {E}$ of the two models. (**c**) The comparison of lithospheric thickness evolution at the center in the CTM (thick solid line), Model 22 (thin solid line), and Model 12 (dashed line). (**d**–**g**) Viscosity evolution of CTM at 8,9,10, and 10.8 Ma with isotherms at 773, 1273, and 1613 K. The 1613 K isotherm is identified with the thermal lithosphere-asthenosphere boundary. The Moho is represented by yellow solid line. (**h**–**k**) Same as (**d**–**g**) but for Model 22 at 9,10,12, and 20 Ma.

The constant traction model (CTM) with $\tau (t) = \text {ILP} + \tau _{0}$ (Fig. 1a) reached continental breakup while model 22 with $\tau (t; 8, 120, 4)$ (Fig. 1a) did not.

In the CTM, $V_\text {E}$ increased from 1.8 mm/yr at 7 Ma to 16 mm/yr by 9 Ma (Fig. 1b). Continental breakup (CB) occurred in this model at 10.8 Ma, when $V_\text {E}$ reached 94 mm/yr. In contrast, $V_\text {E}$ in Model 22 started decreasing as soon as the traction reduction initiated at 8 Ma ($t_i$) ( Fig. 1b). After this 0.5 Ma-long period of deceleration, $V_\text {E}$ began increasing again until 9.1 Ma to about 6 mm/yr (Fig. 1b). After 9.1 Ma, $V_\text {E}$ transitioned into decreasing phase until the end of $\delta t$ (Fig. 1b) and then remained constant at around 0.2 mm/yr afterwards until the end of the model run.

The temporal change of the lithospheric thickness in the rift zone ($T_{L}$) in the CTM showed a sigmoidal pattern, rapidly decreasing from about 110 km at 8.5 Ma to 15 km by the time of CB at 10.8 Ma (Fig. 1c). While having the same thickness as in the CTM at 8.5 Ma, the lithosphere in Model 22 decreased only to about 90 km by 12.5 Ma, maintaining that thickness afterwards (Fig. 1c) because the rate of continued thinning by the boundary traction was just enough to compensate for the rate of thickening due to cooling. Outside the broadened rift zone with large accumulated plastic strain, the high-strength lithosphere underwent minimal deformation in both models (Fig. 1d,h).

The thinnest part of the crust in the CTM was within the rift zone, being about 2.5 km thick at 10.8 Ma (Fig. 1g, Supplementary Movie 1). Since that thickness is the vertical model resolution in this region, the crust layer has been essentially ruptured by this time. The corresponding crustal stretching factor ($\beta _\text {crust}$), the ratio of the original crustal thickness of 40 km to this thickness, was 16. In Model 22, $\beta _\text {crust}$ was 2.6 after 20 Ma (Fig. 1k, Supplementary Movie 2).

### 25% traction reduction: $\delta \tau =40$ MPa

For the 13 models (Model 1–13 in Table 2) subjected to 25 % of traction reduction, continental breakup was observed to occur with smaller reduction rates ($\delta \tau / \delta t$) and later initiation of traction reduction (i.e., greater $t_i$).

#### Models with $t_{i}$ = 4 Ma

**Fig. 2:**
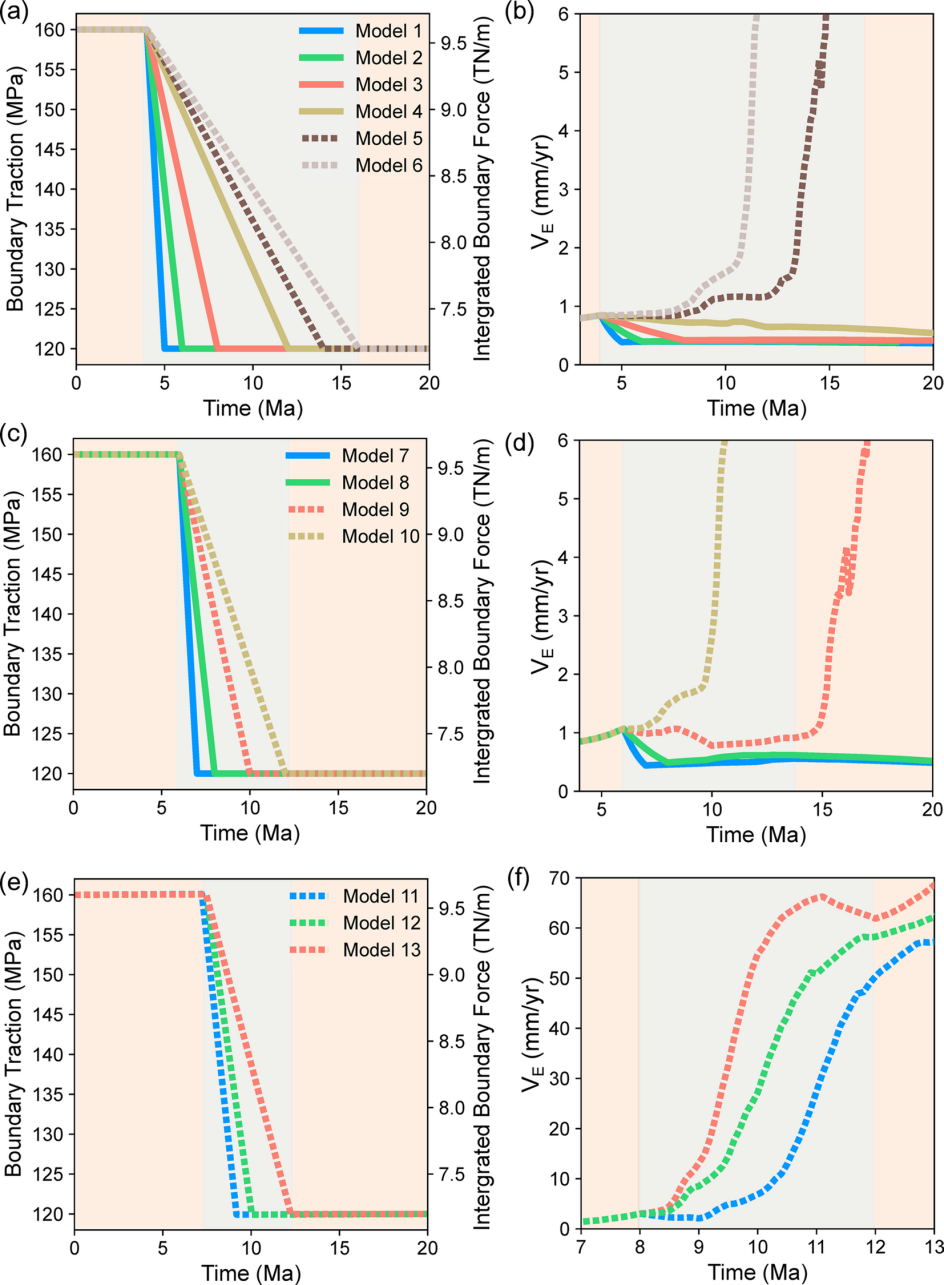
(**a**) Boundary traction magnitudes and (**b**) $V_\text {E}$ plotted against time for the models with for $\delta \tau$ = 40 MPa and $t_\text {i}$ = 4 Ma. FR models are represented with solid lines while models that reach CB denoted by dashed lines. (**c,d**) Same as (**a,b**) but for the models with $t_\text {i}$ = 6 Ma. (e,f) Same as (**a,b**) but for the models with $t_\text {i}$ = 8 Ma.

Model 1 to Model 3 contained $t_{i}$ = 4 Ma but their $\delta t$ values were 1, 2 and 4 Ma (Fig. 2a). $V_\text {E}$ linearly decreased during $\delta t$, remained constant at around 0.5 mm/yr after $\delta t$ (Fig. 2b). With the slowest traction reduction rate ($\delta \tau /\delta t$) among the four FR models, $V_\text {E}$ in Model 4 decreased at the slowest rate almost linearly from 0.9 mm/yr at $t_{i}$ to 0.6 mm/yr at 20 Ma. In these FR models, the crust and lithosphere maintained a significant portion of their initial values by 20 Ma in the rift zone.

Model 5’s $V_\text {E}$ overall increased during the force reduction period, 4 to 14 Ma (Fig. 2a). After 14 Ma, $V_\text {E}$ began increasing at a much greater rate (Fig. 2b, Supplementary Movie 3) reaching 53 mm/yr and completing CB at 17.6 Ma. In Model 6, $V_\text {E}$ also increased during the force reduction period, from 4 to 16 Ma (Fig. 2a). However, in contrast to Model 5, $V_\text {E}$ began increasing at a much greater rate in the middle of that period (Fig. 2b, Supplementary Movie 4), reaching 57 mm/yr at 14.1 Ma. By this time, CB completed in this model.

#### Models with $t_{i}$ = 6 Ma

Models 7 and 8 with $\delta t$ = 1 and 2 Ma (Fig. 2c) did not complete continental breakup whereas Model 9 and 10 with greater $\delta t$ of 4 and 6 Ma (Fig. 2c) did. Model 7 and Model 8 showed reduction in $V_\text {E}$ during $\delta t$ from 1.1 mm/yr at 6 Ma to 0.4 mm/yr after $\delta t$ (Fig. 2d, Supplementary Movie 5). In Model 9, $V_\text {E}$ decreased from 1.1 mm/yr at 6 Ma to 0.7 mm/yr by the end of it’s $\delta t$. While the boundary traction magnitude is fixed at 120 MPa (as for all models in this section) after 10 Ma, $V_\text {E}$ slowly increased until 15 Ma and with a much greater rate afterwards, completing CB by 19.7 Ma with $V_\text {E}$ of a 55 mm/yr. Model 10 with a slower traction reduction rate ($\delta t$ = 6 Ma) showed continuously increasing $V_\text {E}$ during the traction reduction period reaching 6 mm/yr by 10 Ma (Fig. 2d, Supplementary Movie 6). CB occurred in Model 10 at 13.5 Ma with $V_\text {E}$ reaching 56 mm/yr.

#### Models with $t_{i}$ = 8 Ma

All of Models 11-13, with $t_i$ of 8 Ma and $\delta t$ of 1, 2 and 4 Ma (Fig. 2e), reached CB (Fig. 2f, Supplementary Movie 7, Movie 8, and Movie 9). Only Model 11 showed a slight reduction in $V_\text {E}$ during $\delta t$ but $V_\text {E}$ started increasing after $\delta t$ reaching 55 mm/yr at 12.6 Ma. In Model 12 and 13, $V_\text {E}$ continued increasing throughout $\delta t$ reaching 56 mm/yr at 11.7 Ma, and 65 mm/yr at 11.2 Ma.

### 50% force reduction: $\delta \tau =80$ MPa

Rifting failed in almost all the models (Models 14–20) in this group, where the boundary traction magnitude was reduced by 50% (i.e., $\delta \tau =$ 80 MPa). Model 21 was the single exception that reached CB, in which the traction reduction began latest (i.e., $t_i = 8$ Ma) and was relatively slow (i.e., $\delta t = 4$ Ma, Supplementary Movie 11).

**Fig. 3:**
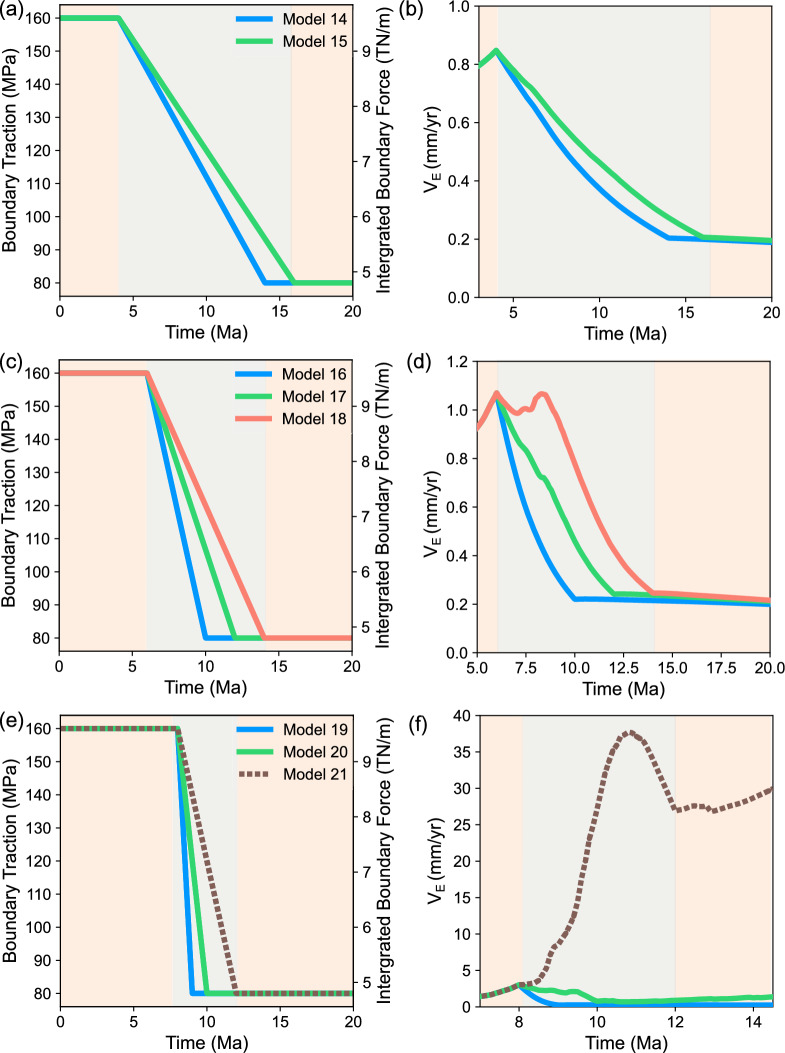
(**a**) Boundary traction magnitudes and (**b**) $V_\text {E}$ plotted against time for the models with for $\delta \tau$ = 80 MPa and $t_\text {i}$ = 4 Ma. (**c,d**) Same as (**a,b**) but for the models with $t_\text {i}$ = 6 Ma. (**e,f**) Same as (**a,b**) but for the models with $t_\text {i}$ = 8 Ma. FR models are represented with solid lines while models that reach CB denoted by dashed lines.

$V_\text {E}$ generally decreased as the boundary traction magnitude did: e.g., Model 14-17 and 19 (Fig. 3b,d,f). However, with a certain combination of the start time and rate of traction reduction as in Model 18 (Fig. 3d), $V_\text {E}$ did not monotonically decreases but rebounded during the first half of the traction reduction period, 8-9 Ma. Such a velocity rebound was also seen in Model 20 but not early in the traction reduction period but later between 9-9.5 Ma during its $\delta t$ (Fig. 3f, Supplementary Movie 10).

We summarized all the model results in Table 1 in terms of the CB criterion and other quantities used in this section. Also, Supplementary Figure 1 shows the viscosity evolution in Model 5 and 9; and Supplementary Figure 2 shows those of Model 21 and 10. The lithospheric thickness evolution in all the models showin in Fig. 2 and 3 can be found in Supplementary Figure 3 and 4, respectively.

**Table 1:** Model-derived values of the crustal stretch factor ($\beta _{\text {crust}}$), the final lithospheric thickness ($T_{L}$), $V_\text {E}$, the timing for continental breakup ($t_{\text {CB}}$), end state marked as whether CB or FR, and $\Delta I_n$ (see the text for definition).

Name	$\beta _{\text {crust}}$	$T_{L}$ (km)	$V_\text {E}$ (mm/yr)	$t_{CB}$ (Ma)	CB($\bigcirc$)/FR($\times$)	Δ*I*_n_ (TN Ma/m)
Model 1	1.0	139	0.4	-	$\times$	373.2
Model 2	1.1	129	0.4	-	$\times$	361.2
Model 3	1.1	128	0.4	-	$\times$	337.2
Model 4	1.3	124	0.55	-	$\times$	289.2
Model 5	16	15	53	17.6	$\bigcirc$	265.2
Model 6	16	15	57	14.1	$\bigcirc$	241.2
Model 7	1.2	125	0.5	-	$\times$	325.2
Model 8	1.3	124	0.5	-	$\times$	313.2
Model 9	16	15	51	19.7	$\bigcirc$	289.2
Model 10	16	15	56	13.5	$\bigcirc$	265.2
Model 11	16	15	55	12.6	$\bigcirc$	277.2
Model 12	16	15	56	11.7	$\bigcirc$	265.2
Model 13	16	15	65	11.2	$\bigcirc$	241.2
Model 14	1.1	129	0.2	-	$\times$	530.4
Model 15	1.1	128	0.2	-	$\times$	482.4
Model 16	1.1	128	0.2	-	$\times$	578.4
Model 17	1.2	128	0.2	-	$\times$	530.4
Model 18	1.3	124	0.2	-	$\times$	482.4
Model 19	1.3	124	0.2	-	$\times$	554.4
Model 20	2.6	91	2.6	-	$\times$	530.4
Model 21	16	15	27	12.5	$\bigcirc$	482.4
Model 22	2.6	97	0.2	-	$\times$	723.6

## Discussion

Our models demonstrate that later initiation of traction reduction (greater $t_{i}$) and slower rates of reduction (smaller $\delta \tau /\delta t$) promote continental breakup (CB). Specifically, if traction reduction begins when the lithosphere has weakened to a critical threshold, CB can occur even with diminishing boundary traction. Given that our study assumed an initially large boundary traction sufficient for CB, it follows that CB is further promoted when the traction remains near this high value for an extended period during a slow reduction.

The following examples clearly illustrate these findings. Rifting failed in Models 1 and 7, both of which had a traction reduction rate of 40 MPa/Ma. Although traction reduction in Model 7 began 2 Ma later than in Model 1, the final outcome remained unchanged. In contrast, Model 11, which also had a 40 MPa/Ma reduction rate, achieved continental breakup (CB). This occurred because traction reduction in Model 11 began 4 Ma later than in Model 1 and 2 Ma later than in Model 7. Furthermore, an examination of models sharing the same $t_{i}$ in Figs. 2 and 3 reveals that earlier initiation of traction reduction does not invariably result in failed rifting (FR) but sufficiently slow traction reduction can promote CB.

The absolute magnitude of traction reduction significantly influenced the rift system’s final state as expected. With a 50% reduction from the initial boundary traction, all models except Model 21 failed to achieve continental breakup (CB) (Fig. 3). This occurred despite some of these models sharing identical $t_{i}$ and $\delta \tau /\delta t$ values with successful CB models in Fig. 2. Even in Model 21, which did achieve CB, $V_\text {E}$ exhibited a notable decrease during the final 1 Ma of traction reduction (11–12 Ma) (Fig. 3f). This reduction in $V_\text {E}$ indicates a slowing of the stretching process, suggesting that while CB was ultimately achieved, the traction reduction magnitude impacted the rate of extension.

**Fig. 4:**
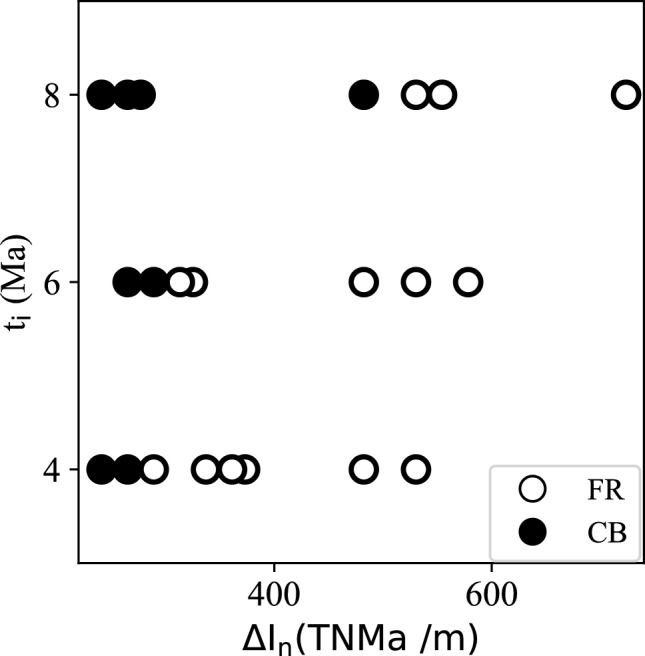
The final state of the models as a function of their $\Delta I_{n}$ and $t_i$ values.

The FR and CB models are clearly separated in the plot (Fig. 4) such that CB is associated with the smaller impulse differences for a given $t_{i}$. Also, the greater $t_{i}$ tend to allow CB for the models with the greater impulse differences. The non-linear boundary between CB and FR reflects that the lithospheric weakening is not a linear process in time.

Non-monotonic evolution of $V_\text {E}$ seen in our models can be understood in the light of the following force balance:

1 $$\begin{aligned} F_{B}(t) = F_{L}(V_{\text {E}},t) + F_{A}(V_{\text {E}},t), \end{aligned}$$

where $F_{B}$ is the depth-integrated deviatoric boundary traction, $F_{L}$ is the depth-integrated lithospheric strength, and $F_{A}$ is the force required for viscous shear flow in asthenosphere (see Appendix A for full details). During an early phase of rifting, lithosphere is still strong and thus $V_\text {E}$ is small, a few mm/yr. In other words, $F_{A}$ is small in magnitude and thus $F_{B}$ is mostly balanced with the strength of lithosphere, $F_{L}$. As rifting progresses and the rift zone in the lithosphere gets weaker thermally and mechanically, $F_{L}$ rapidly decreases. The assumed force balance then leads to rapid increase in $V_\text {E}$ such that $F_{A}$ becomes a significant component in the force balance. This is the $V_\text {E}$ evolution path our CTM model showed (Fig. 1b) and corresponds to the “speedup before breakup” observed in many passive margins^35^.

**Fig. 5:**
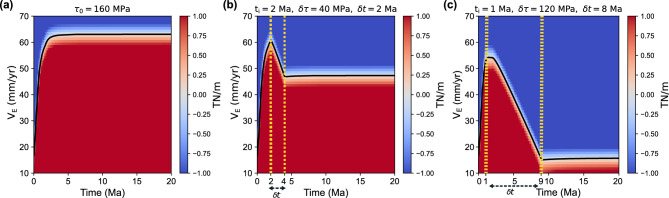
The residual force, $F_{B}-(F_{L}+F_{A}$), as a function of time and $V_{\text {E}}$, acquired as described in Appendix A for (**a**) a constant boundary traction ($\tau _{0}$) of 160 MPa and (**b**) a time-dependent case that reach CB as $\tau (t; 2 \text { Ma}, 40 \text { MPa}, 2 \text { Ma})$, and (**c**) a failed rift scenario with $\tau (t; 1 \text { Ma}, 120 \text { MPa}, 8 \text { Ma})$. Solid black lines mark the $V_\text {E}$ values at a given time that satisfy the force balance.

Our semi-analytic solution to the force balance equation (see Appendix A) further indicates that when $F_{B}$ is constant and finite, $V_\text {E}$ is eventually regulated to reach a steady-state value because $F_{L}$ becomes less significant and $F_{A}$, increasing with $V_\text {E}$, should balance with the constant $F_{B}$. In our calculation with $\tau _{0}$ = 160 MPa, CB was completed and $V_{E}$ reached a steady-state value of 63 mm/yr after 4 Ma (Fig. 5a). The constant speed after CB is reasonable because the post-CB force balance in the analytic model corresponds to that of the seafloor spreading.

The steady-state $V_{E}$ reached in the CB models should be understood as representing a seafloor spreading rate, not the rifting rate. However, some steady-state $V_{E}$ values approach or even exceed the fastest end of the known seafloor spreading rate spectrum: e.g., 63 mm/yr in this constant-traction analytic model and and 94 mm/yr in the CTM (Fig. 1). There are two contributing factors to this result. One is the constant traction of 160 MPa. A smaller value would have lowered the steady-state extension rate. In fact, $V_{E}$ values at the timing of CB in all the models with reduced tractions were consistently much smaller, $\le$ 65 mm/yr. The other factor is the technical limitations in our modeling approach. For instance, in the current setting, the far end of continental lithosphere from the rift zone continuously move out of the model domain. Those portions cannot contribute to the force balance, effectively lowering the resisting forces.

When $F_{B}$ decreases over time as assumed in this study, $V_\text {E}$ will arrive at a steady-state value after passing a greater peak value. In our calculation with $t_{i} = \delta t =$ 2 Ma and $\delta \tau =$40 MPa, the peak value was 61 mm/yr and the steady-state value was 48 mm/yr (Fig. 5b). With a greater traction reduction of 120 MPa, the semi-analytic model reproduced the pattern of $V_{E}$ evolution in most of the FR models (Fig. 5c), which is characterized by the monotonic decrease since $t_{i}$ and during $\delta t$ followed by a steady-state low $V_{E}$. However, $V_{E}$ magnitudes are overall greater in the semi-analytic model due to the differences in the model setup.

Caution is needed when prospecting a rift system’s ultimate state based on its present-day rifting rate. Even as driving force magnitude is monotonically decreasing in an FR model, the rifting rate (i.e., $V_\text {E}$) can temporarily increase as in Model 18 (Fig. 3d) and Model 22 (Fig. 1b). Model 9, on the other hand, showed a prolonged period of a very small rifting rate of 1 mm/yr throughout its entire $\delta t$, 6–14 Ma (Fig. 2d). However, rather than losing to the lithosphere strengthening effects, rifting started accelerating around 15 Ma eventually reaching CB. This behavior of Model 9 suggest that with the slow spreading rates in Rhine Graben^37^ and Rio Grande rift^38^ alone, reported to be around 1 mm/yr, we cannot rule out the possibility that these rift systems might get invigorated in the future. What matters is the balance between the current lithospheric strength and the available driving force.

## Methods

### Governing equations

We construct two-dimensional (2D) continental extension models using the open source code ASPECT^39,40^. In this study, velocity and pressure are solved for using the incompressible Boussinesq approximation, where the continuity and momentum balance equations are given by

2 $$\begin{aligned} \nabla {\cdot \textbf{u}}&=0, \end{aligned}$$

3 $$\begin{aligned} -\nabla \cdot (2\,\mu \,\dot{\varvec{\varepsilon }}) + \nabla p&=\rho \,\textbf{g}, \end{aligned}$$

where $\textbf{u}$ is the velocity vector, $\mu$ is the dynamic viscosity, $\dot{\varvec{\varepsilon }}$ is the deviatoric strain rate, *p* is the pressure, $\rho$ is the density, $\textbf{g}$ is the gravitational acceleration.

Thermal evolution is modeled through the advection-diffusion equation:

4 $$\begin{aligned} \rho C_p \left( \frac{\partial T}{\partial t} + \textbf{u} \cdot \nabla T \right) -\nabla \cdot k\nabla T&= \rho H \end{aligned}$$

$C_p$ is the specific heat capacity at constant pressure, $T$ is the temperature, $t$ is time, $k$ is the thermal conductivity, and $H$ is the internal heating rate.

The density variation follow the Boussinesq approximation:

5 $$\begin{aligned} \rho&= \rho _0(1-\alpha (T-T_0)), \end{aligned}$$

where $\rho _\text {0}$ is the reference density, $\alpha$ is the thermal expansivity, and $T_\text {0}$ is the reference temperature.

To simulate brittle behaviors, we use a viscoplastic rheology with strain weakening. The viscous flow model takes the harmonic mean of the viscosities derived from diffusion and dislocation creep^41^ such that the effective viscosity is defined as

6 $$\begin{aligned} \eta _{\text {eff}} = \left( \frac{1}{\eta _{\text {eff}}^\text {diff}}+ \frac{1}{\eta _{\text {eff}}^\text {dis}}\right) ^{-1}, \end{aligned}$$

where

7 $$\begin{aligned} \eta _{\text {i}} = \frac{1}{2} A^{-\frac{1}{n_i}} d^\frac{m_i}{n_i} \dot{\varepsilon _i}^{\frac{1-n_i}{n_i}} \exp \left( \frac{E_i^*+ PV_i^*}{n_iRT}\right) \end{aligned}$$

where *i* corresponds to diffusion or dislocation creep, $A_i$ are the prefactors, *d* is the grain size, $\dot{\varepsilon }$ is the square root of the second invariant of the strain rate tensor, $n_i$ and $m_i$ are the stress and grain size exponents, *P* is the pressure, $E_i$ are the activation energies, $V_i$ are the activation volumes, *R* is the gas constant, and *T* is the temperature.

Brittle (plastic) behavior follows a Drucker-Prager yield criterion formulation, where the yield stress in 2-D is a function of the cohesion ($C$), angle of internal friction ($\phi$), and pressure ($P$):

8 $$\begin{aligned} \sigma _y = P \sin (\phi ) + C \cos (\phi ) \end{aligned}$$

### Model setup

**Fig. 6:**
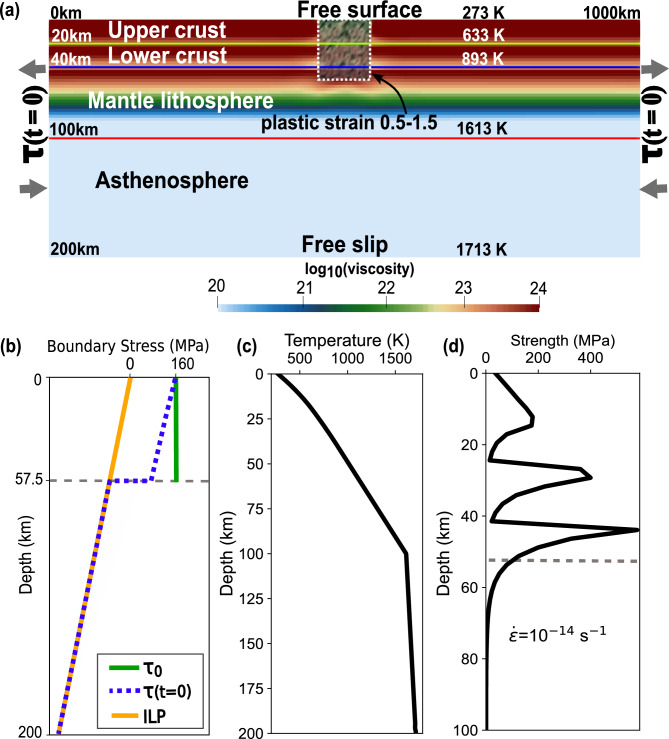
(**a**) The model domain 1000 km in x direction and 200 km in y direction, with three compositional layering (upper crust, lower crust, and mantle). The mantle composition is divided into mantle lithosphere (40-100 km), and asthenosphere (100–200 km) as a thermal boundary where inflow of material allowed through side walls below the mantle lithosphere. The dashed box represents the initial weak zone with random plastic strain values between 0.5 and 1.5. The background is the viscosity distribution. (**b**) The applied boundary traction $\tau (t=0)$ (dashed blue line) to the left and right side boundaries of the models. $\tau (t=0)$ is the resultant of adding the initial Lithostatic pressure (ILP) (solid orange line) to deviatoric traction $\tau _\text {0}$ (solid green line). (**c**) The initial depth distribution of temperature. (**d**) The yield strength envelop (i.e., differential stress) for the assumed rheologies for the assumed uniformed extensional strain rate of 10$^{-14}$ 1/s.

Our 2D models have a width of 1000 km and a thickness of 200 km (Fig. 6a). The domain is discretized into square elements of a size of 2.5 km above 100 km depth and 5 km below.

The model domain contains three compositional layers: Upper crust (0–20 km depth), lower crust (20–40 km), and mantle (40–200 km) (Fig. 6a). The upper crust and lower crust follow dislocation creep flow laws for wet quartzite^42^ and wet anorthite^43^. For mantle, we assume dry olivine^25^, that behaves as a composite between dislocation and diffusion creep^44^.We adopted these commonly assumed compositions although they are substantial simplifications. For instance, rheological behavior of continental lithosphere is highly sensitive to its depth-dependent water content [e.g.,^45,46^].

The initial friction angle is set to be 30$^{\circ }$ and the initial cohesion either 20 or 80 MPa as further described below. We employ a strain weakening rule such that both of these parameters are linearly reduced to 1% of the initial values as the square root of the second invariant of plastic strain increases.

A weak zone of 50 km by 50 km (dashed box in Fig. 6a) is placed in the top center of a model to promote focused extension. The weak zone starts with a randomized distribution of plastic strain between 0.5 and 1.5^47^. To remove the sensitivity to the initial plastic strain distribution, we use one instance of random plastic strain distribution for all the models. All the layers within the top 50 km gets an initial cohesion of 80 MPa except the weak zone, where the initial cohesion is 20 MPa. This assignment of initial cohesion values is needed for suppressing boundary deformation and nonphysical large velocity fluctuations near the top left and right corners of the model domain.

The bottom boundary is set to be free-slip, and the top boundary is a free surface^48^, which is advected using both the vertical and horizontal components of the velocity field.

Tractions applied on the left and right boundaries are the sum of initial lithostatic pressure ($P_{0,\text {lith}}$) and deviatoric traction ($\tau _\text {0}$) (Fig. 6b). The value $\tau _\text {0}$ is only assigned for the top 57.5 km (Fig. 6b) along either left or right boundaries, which represent the high-viscosity ($\ge 1.1 \times 10^{23}$ Pa$\cdot$s) portion of the lithosphere. The deviatoric components are time-dependent, rendering the entire traction boundary time-dependent, where we reduce the assigned traction over time to understand under what conditions of traction reduction could lead to either continental breakup or a failed rift system. The time-dependent traction (TDT) is denoted as $\tau (t)$ and defined as

9 $$\begin{aligned} \tau (t; t_i, \delta \tau , \delta t) = P_{0,\text {lith}} + {\left\{ \begin{array}{ll} \tau _{0} & \text {if }\quad t< t_{i}, \\ \tau _{0} - \frac{\delta \tau }{\delta t} (t - t_i) & \text {if } t_{i} \le t < t_{i} + \delta t, \\ \tau _{0} - \delta \tau & \text {if }\quad t \ge t_i + \delta t, \end{array}\right. } \end{aligned}$$

where $t_i$ is the start time of the reduction, $\delta \tau$ is the amount of magnitude reduction, $\delta t$ is the period of reduction and $\tau _{0}$ is the initial deviatoric traction. The value of $\tau _{0}$ is fixed at 160 MPa for all of our models, which is equivalent to $9.6\times 10^{12}$ $\hbox {Nm}^{-1}$ and compatible with the value range suggested by^31^ for 55 $\hbox {mWm}^{-2}$ surface heat flux. Run for 20 Ma of model time, all the TDT models created for this study are listed in Table 2.

**Table 2:** List of all the time-dependent models and parameters for $\tau (t)$ given in Eq. (9).

Name	$t_i$(Ma)	$\delta \tau$(MPa)	$\delta t$(Ma)	Name	$t_i$(Ma)	$\delta \tau$(MPa)	δ_t_ (Ma)
Model 1	4	40	1	Model 12	8	40	2
Model 2	4	40	2	Model 13	8	40	4
Model 3	4	40	4	Model 14	4	80	10
Model 4	4	40	8	Model 15	4	80	12
Model 5	4	40	10	Model 16	6	80	4
Model 6	4	40	12	Model 17	6	80	6
Model 7	6	40	1	Model 18	6	80	8
Model 8	6	40	2	Model 19	8	80	1
Model 9	6	40	4	Model 20	8	80	2
Model 10	6	40	6	Model 21	8	80	4
Model 11	8	40	1	Model 22	8	120	4

Following^49^, we set up an initial geotherm (Fig. 6c) for continental lithosphere with thermal conductivity of 2.5 $\hbox {Wm}^{-1}$ $\hbox {K}^{-1}$, and surface temperature of 273 K, and a surface heat flow of 55 $\hbox {mWm}^{-2}$, and constant radiogenic heating rates in each compositional layer. The top and bottom temperatures are fixed at 273 and 1713 K while side boundaries are assumed to be insulating. With the initial geotherm, assumed rheologies and material properties listed in Supplementary Table 1, we get the initial strength profile for the strain rate of 10$^{-14}$ $\hbox {s}^{-1}$ shown in Fig. 6d.

The mean extension velocity ($\text {V}_\text {E}$) is extensively used for the presentation of the model results and their analysis. $\text {V}_\text {E}$ is calculated at 0.1 Ma intervals, using the x-component of velocity from the top-surface nodes on the right half of the model domain.

We considered CB to have occurred in a model when the crust in the rift zone became thinner than 2.5 km, the size of an element in the rift zone, for the first time. The corresponding crustal stretching factor ($\beta _\text {crust}$), defined as the ratio of the original crustal thickness of 40 km to this thickness, is 16. When CB did not occur in a model, we labeled the model FR (Failed Rift).

### Time-integrated boundary force

To quantitatively assess the effect of traction reduction on continental breakup, we define the difference in boundary impulse ($\Delta I_n$) between a given model and CTM. This parameter serves to represent the combined influence of the traction reduction parameters: $t_i,\delta t,$ and $\delta \tau$. We define $\Delta I_n$ as:

10 $$\begin{aligned} \Delta I_{n} = \int _{t_{i}}^{20 Ma}\int _{0}^{57.5 km}(\tau _{0} - \tau _{n}) dz\,dt, \end{aligned}$$

where $\tau _{n}$ is the model’s boundary traction magnitude after $t_{i}$. Because the side boundaries are normal to the *x* axis, $\tau _{n}$ is equivalent to $\tau _{xx}$ in practice. The depth integration limit is 57.5 km because we only assign $\tau _0$ to the part of lithosphere as shown in Fig. 6b. The calculated $\Delta I_{n}$ values are listed in Table 1 and plotted with $t_i$ for all the models in Fig. 4.

## Conclusions

This study investigated the influence of temporally varying boundary tractions on continental rift evolution, revealing critical factors governing the transition from rifting to continental breakup (CB) or failed rift (FR). Our numerical models demonstrated that later initiation of traction reduction ($t_i$) and slower reduction rates ($\delta \tau /\delta t$) significantly promote CB. This is attributed to the prolonged maintenance of high driving forces, allowing for substantial lithospheric weakening before traction reduction begins. As expected, the absolute magnitude of traction reduction ($\delta \tau$) played a pivotal role, too. A 50% reduction almost invariably led to FR, emphasizing the necessity of maintaining sufficient driving force to overcome lithospheric resistance. However, a 25% reduction allowed for CB, particularly with optimized initiation times and reduction rates.

Our models also revealed non-monotonic evolution of extension velocities ($V_\text {E}$), demonstrating that even during periods of decreasing driving force, rifting rates can temporarily increase. This behavior is explained by the dynamic force balance between boundary tractions, lithospheric strength, and asthenospheric resistance. Furthermore, our semi-analytical solutions showed that $V_\text {E}$ approaches a steady-state value when boundary tractions are constant or decreasing. Finally, our results caution against relying solely on present-day rifting rates to predict a rift system’s ultimate fate because currently slow rifting can still undergo future acceleration ultimately reaching CB.

## Supplementary Information

Supplementary Information 1.

Supplementary Information 2.

Supplementary Information 3.

Supplementary Information 4.

Supplementary Information 5.

Supplementary Information 6.

Supplementary Information 7.

Supplementary Information 8.

Supplementary Information 9.

Supplementary Information 10.

Supplementary Information 11.

Supplementary Information 12.

Supplementary Information 13.

## Data Availability

The ASPECT parameter files for each of the 23 model runs, along with Jupyter notebooks for the analytical solution and velocity extraction, are archived and publicly available via Figshare at https://doi.org/10.6084/m9.figshare.28590731.v2. The ASPECT code used in this study is open source and can be downloaded from https://aspect.geodynamics.org/.

## References

[1] Ojakangas, R., Morey, G. & Green, J. The mesoproterozoic midcontinent rift system, Lake Superior Region, USA. *Sed. Geol.* **141–142**, 421–442. 10.1016/S0037-0738(01)00085-9 (2001).

[2] Stein, S. et al. Insights from North America’s failed Midcontinent Rift into the evolution of continental rifts and passive continental margins. *Tectonophysics* **744**, 403–421. 10.1016/j.tecto.2018.07.021 (2018).

[3] Hildenbrand, T. G. Rift Structure of the Northern Mississippi Embayment from the analysis of gravity and magnetic data. *J. Geophys. Res.* **90**, 12607. 10.1029/JB090iB14p12607 (1985).

[4] Cox, R. T. & Van Arsdale, R. B. The Mississippi Embayment, North America: a first order continental structure generated by the Cretaceous superplume mantle event. *J. Geodyn.* **34**, 163–176. 10.1016/S0264-3707(02)00019-4 (2002).

[5] Ghomsi, F. E. K., Tenzer, R., Njinju, E. & Steffen, R. The crustal configuration of the West and Central African Rift System from gravity and seismic data analysis. *Geophys. J. Int.* **230**, 995–1012. 10.1093/gji/ggac089 (2022).

[6] Fairhead, J. Mesozoic plate tectonic reconstructions of the central South Atlantic Ocean: the role of the West and Central African rift system. *Tectonophysics* **155**, 181–191. 10.1016/0040-1951(88)90265-X (1988).

[7] Thomas, W. A mechanism for tectonic inheritance at transform faults of the Iapetan margin of Laurentia. *Geosci. Can.* **41**, 321–344 (2014).

[8] Huerta, A. D. & Harry, D. L. The transition from diffuse to focused extension: modeled evolution of the West Antarctic Rift system. *Earth Planet. Sci. Lett.* **255**, 133–147. 10.1016/j.epsl.2006.12.011 (2007).

[9] Hall, J., Wilson, T. & Henrys, S. *Structure of the central Terror Rift, western Ross Sea* (Antarctica. Tech, Rep, 2007).

[10] Voorde, M., Færseth, R. B., Gabrielsen, R. H. & Cloetingh, S. A. P. L. Repeated lithosphere extension in the northern Viking Graben: a coupled or a decoupled rheology?. *Geol. Soc. Lond. Special Publ.* **167**, 59–81. 10.1144/GSL.SP.2000.167.01.04 (2000).

[11] Underhill, J. R. & Partington, M. A. Jurassic thermal doming and deflation in the North Sea: implications of the sequence stratigraphic evidence. *Geol. Soc. Lond. Petrol. Geol. Conf. Ser.* **4**, 337–345. 10.1144/0040337 (1993).

[12] Torres-Acosta, V. et al. Cenozoic extension in the Kenya Rift from low-temperature thermochronology: Links to diachronous spatiotemporal evolution of rifting in East Africa. *Tectonics* **34**, 2367–2386. 10.1002/2015TC003949 (2015).

[13] Heine, C., Zoethout, J. & Müller, R. D. Kinematics of the South Atlantic rift. *Solid Earth* **4**, 215–253. 10.5194/se-4-215-2013 (2013).

[14] Brune, S. et al. Geodynamics of continental rift initiation and evolution. *Nat. Rev. Earth . Env.*10.1038/s43017-023-00391-3 (2023).

[15] Forsyth, D. & Uyeda, S. On the relative importance of the driving forces of plate motion. *Geophys. J. Int.* **43**, 163–200. 10.1111/j.1365-246X.1975.tb00631.x (1975).

[16] Capitanio, F., Morra, G. & Goes, S. Dynamic models of downgoing plate-buoyancy driven subduction: subduction motions and energy dissipation. *Earth Planet. Sci. Lett.* **262**, 284–297. 10.1016/j.epsl.2007.07.039 (2007).

[17] Royden, L. H. & Husson, L. Trench motion, slab geometry and viscous stresses in subduction systems. *Geophys. J. Int.* **167**, 881–905. 10.1111/j.1365-246X.2006.03079.x (2006).

[18] Funiciello, F., Morra, G., Regenauer-Lieb, K. & Giardini, D. Dynamics of retreating slabs: 1. Insights from two-dimensional numerical experiments. *J. Geophys. Res. Solid Earth* **108**, 2001JB000898. 10.1029/2001JB000898 (2003).

[19] Gerya, T. V., Yuen, D. A. & Maresch, W. V. Thermomechanical modelling of slab detachment. *Earth Planet. Sci. Lett.* **226**, 101–116. 10.1016/j.epsl.2004.07.022 (2004).

[20] Duretz, T., Gerya, T. V. & May, D. A. Numerical modelling of spontaneous slab breakoff and subsequent topographic response. *Tectonophysics* **502**, 244–256. 10.1016/j.tecto.2010.05.024 (2011).

[21] Burkett, E. R. & Billen, M. I. Three-dimensionality of slab detachment due to ridge-trench collision: laterally simultaneous boudinage versus tear propagation. *Geochem. Geophys. Geosyst.* **11**, 2010GC003286. 10.1029/2010GC003286 (2010).

[22] Duretz, T., Schmalholz, S. M. & Gerya, T. V. Dynamics of slab detachment. *Geochem. Geophys. Geosyst.* **13**, 2011GC004024. 10.1029/2011GC004024 (2012).

[23] Kirby, S. H. & Kronenberg, A. K. Rheology of the lithosphere: selected topics. *Rev. Geophys.* **25**, 1219–1244. 10.1029/RG025i006p01219 (1987).

[24] Karato, S.-I. & Wu, P. Rheology of the upper mantle: a synthesis. *Science* **260**, 771–778. 10.1126/science.260.5109.771 (1993). 17746109 10.1126/science.260.5109.771

[25] Hirth, G. & Kohlstedf, D. Rheology of the upper mantle and the mantle wedge: a view from the experimentalists. *Geophys. Monogr. Am. Geophys. union* **138**, 83–106 (2003).

[26] Kusznir, N. J. Lithosphere response to externally and internally derived stresses: a viscoelastic stress guide with amplification. *Geophys. J. Int.* **70**, 399–414. 10.1111/j.1365-246X.1982.tb04974.x (1982).

[27] Kusznir, N. J. & Park, R. G. Intraplate lithosphere deformation and the strength of the lithosphere. *Geophys. J. Int.* **79**, 513–538. 10.1111/j.1365-246X.1984.tb02238.x (1984).

[28] Kusznir, N. J. & Park, R. G. The extensional strength of the continental lithosphere: its dependence on geothermal gradient, and crustal composition and thickness. *Geol. Soc. Lond. Spec. Publ.* **28**, 35–52. 10.1144/GSL.SP.1987.028.01.04 (1987).

[29] Kusznir, N. J. The distribution of stress with depth in the lithosphere: thermo-rheological and geodynamic constraints. *Philos. Trans. R. Soc. Lond. Ser. A* **337**, 95–110. 10.1098/rsta.1991.0109 (1991).

[30] Takeshita, T. & Yamaji, A. Acceleration of continental rifting due to a thermomechanical instability. *Tectonophysics* **181**, 307–320. 10.1016/0040-1951(90)90024-3 (1990).

[31] Porth, R. A strain-rate-dependent force model of lithospheric strength. *Geophys. J. Int.* **141**, 647–660. 10.1046/j.1365-246x.2000.00115.x (2000).

[32] Yamasaki, T. & Stephenson, R. Potential role of strain hardening in the cessation of rifting at constant tectonic force. *J. Geodyn.* **47**, 47–62. 10.1016/j.jog.2008.07.001 (2009).

[33] Yamasaki, T. & Stephenson, R. Change in tectonic force inferred from basin subsidence: implications for the dynamical aspects of back-arc rifting in the western Mediterranean. *Earth Planet. Sci. Lett.* **277**, 174–183. 10.1016/j.epsl.2008.10.011 (2009).

[34] Yamasaki, T. & Stephenson, R. Back-arc rifting initiated with a hot and wet continental lithosphere. *Earth Planet. Sci. Lett.* **302**, 172–184. 10.1016/j.epsl.2010.12.009 (2011).

[35] Brune, S., Williams, S. E., Butterworth, N. P. & Müller, R. D. Abrupt plate accelerations shape rifted continental margins. *Nature* **536**, 201–204. 10.1038/nature18319 (2016). 27437571 10.1038/nature18319

[36] Buck, W. R. The role of magma in the development of the Afro-Arabian Rift System. *Geol. Soc. Lond. Spec. Publ.* **259**, 43–54 (2006).

[37] Tesauro, M., Hollenstein, C., Egli, R., Geiger, A. & Kahle, H.-G. Continuous GPS and broad-scale deformation across the Rhine Graben and the Alps. *Int. J. Earth Sci.* **94**, 525–537 (2005).

[38] Berglund, H. T. et al. Distributed deformation across the Rio Grande Rift, Great Plains, and Colorado Plateau. *Geology* **40**, 23–26. 10.1130/G32418.1 (2012).

[39] Heister, T., Dannberg, J., Gassmöller, R. & Bangerth, W. High accuracy mantle convection simulation through modern numerical methods???? II: realistic models and problems. *Geophys. J. Int.* **210**, 833–851. 10.1093/gji/ggx195 (2017).

[40] Kronbichler, M., Heister, T. & Bangerth, W. High accuracy mantle convection simulation through modern numerical methods: high accuracy mantle convection simulation. *Geophys. J. Int.* **191**, 12–29. 10.1111/j.1365-246X.2012.05609.x (2012).

[41] Glerum, A., Thieulot, C., Fraters, M., Blom, C. & Spakman, W. Nonlinear viscoplasticity in ASPECT: benchmarking and applications to subduction. *Solid Earth* **9**, 267–294. 10.5194/se-9-267-2018 (2018).

[42] Rutter, E. & Brodie, K. Experimental grain size-sensitive flow of hot-pressed Brazilian quartz aggregates. *J. Struct. Geol.* **26**, 2011–2023. 10.1016/j.jsg.2004.04.006 (2004).

[43] Rybacki, E., Gottschalk, M., Wirth, R. & Dresen, G. Influence of water fugacity and activation volume on the flow properties of fine-grained anorthite aggregates. *J. Geophys. Res. Solid Earth* **111**, 2005JB003663. 10.1029/2005JB003663 (2006).

[44] Berg, A. P., Keken, P. E. & Yuen, D. A. The effects of a composite non-Newtonian and Newtonian rheology on mantle convection. *Geophys. J. Int.* **115**, 62–78 (1993).

[45] Peslier, A. H., Woodland, A. B., Bell, D. R. & Lazarov, M. Olivine water contents in the continental lithosphere and the longevity of cratons. *Nature* **467**, 78–81. 10.1038/nature09317 (2010). 20811455 10.1038/nature09317

[46] Schettino, A. & Ranalli, G. Ultra-slow transverse waves during continental breakup. *Evol. Earth* **1**, 100009. 10.1016/j.eve.2023.100009 (2023).

[47] Naliboff, J. B., Glerum, A., Brune, S., Péron-Pinvidic, G. & Wrona, T. Development of 3-D rift heterogeneity through fault network evolution. *Geophys. Res. Lett.* **47**, e2019GL086611 (2020).

[48] Rose, I., Buffett, B. & Heister, T. Stability and accuracy of free surface time integration in viscous flows. *Phys. Earth Planet. Inter.* **262**, 90–100. 10.1016/j.pepi.2016.11.007 (2017).

[49] Chapman, D. S. Thermal gradients in the continental crust. *Geol. Soc. Lond. Spec. Publ.* **24**, 63–70. 10.1144/GSL.SP.1986.024.01.07 (1986).

