# Supplementary material for: Continental rifts losing driving forces can still complete breakup
Source: Sci Rep. 2025 Oct 23;15:37023. doi: 10.1038/s41598-025-19691-3 (PMC12549862; doi:10.1038/s41598-025-19691-3)
Supplement: Supplementary file 1 — Supplementary Information 1. [file 41598_2025_19691_MOESM1_ESM.pdf]

Appendix A Analytical Model

The temporal evolution of extension velocity rate ($V_{\mathrm{E}}(t)$) in ours models can be understood as a consequence of this simplified force balance:

$$F_B(t) = F_L(V_{hs}, t) + F_A(V_{hs}, t), \tag{A1}$$

where F_B is the depth-integrated deviatoric boundary traction, F_L is the depth-integrated lithospheric strength, and F_A is the distance-integrated shear stress associated with channel-like flows in asthenosphere. In the following, we treat V_{E} and t as independent variables and try to find their values that satisfy eq. (A1).

F_B is computed as follows:

$$F_B = \int_0^{L_0} \tau_B \, dz = \begin{cases} 160 L_0 & \text{if } \tau_B \text{ is constant at 160 MPa,} \\ (160 + (\delta\tau/\delta t)t)\, L_0 & \text{otherwise,} \end{cases} \tag{A2}$$

where L_0 is the initial lithospheric thickness, 100 km, and the unit is 10^6 N/m.

We assumed that all of the lithospheric stretching is focused on a relatively weak rift zone. The rift zone has an initial width (W_0) of 50 km (Fig. A1). The rift zone width changes over time (t) and its rate of change is proportional to V_{E}:

$$W(V_{\mathrm{E}}, t) = W_0 + 2\,\alpha\, V_{\mathrm{E}}\, t, \tag{A3}$$

where the factor of 2 is introduced because V_{E} is the half-spreading rate and α is a nudge factor for which 0.40 was chosen due to V_{E} ratio of weak zone to the length of the plate. The lithospheric thickness also changes with time and V_{E}. We assume that it accrues an increment from the half-space cooling model from t_0 to t, where $t_0 = (L_0/2.57)^2/\kappa$, the age corresponding to L_0 according to the same cooling model and a given thermal diffusivity κ; then thinned by a time-dependent factor of $\beta(V_E, t) = W(V_E, t)/W_0$. Under these assumptions, the lithospheric thickness is given as

$$L(V_{\mathrm{E}}, t) = \frac{1}{\beta(V_E, t)} \left[L_0 + 2.32 \left(\sqrt{\kappa(t_0 + t)} - \sqrt{\kappa t_0} \right) \right]. \tag{A4}$$

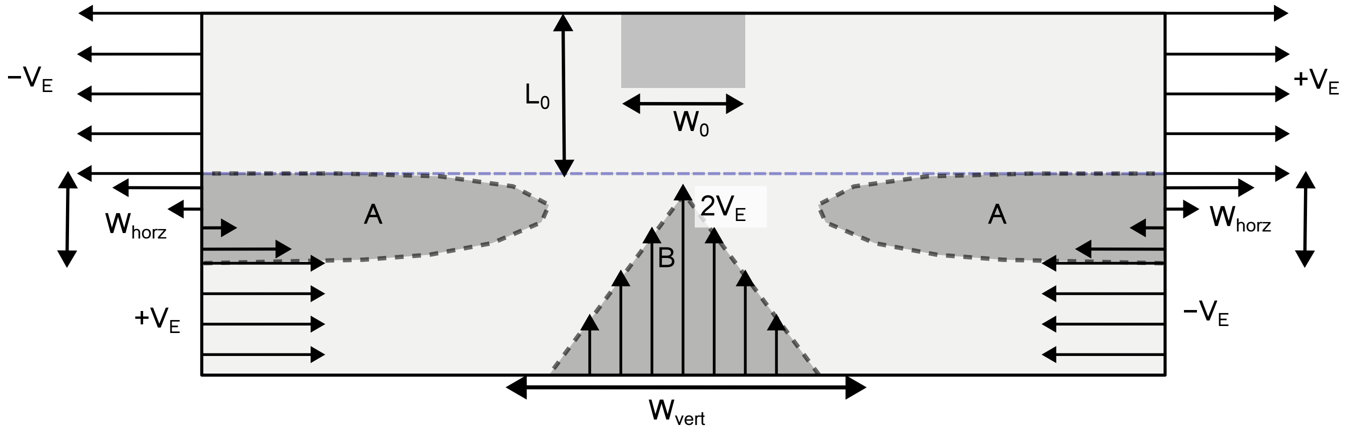

Figure A1: Illustration describing the setup for the semi-analytic model.

F_L can increase or decrease over time depending on whether strengthening by cooling or weakening by thinning is dominant. Here, we considered only the latter case. In the former case, V_{E} would simply decrease over time (e.g., Model 1 to Model 4 in Fig. 3b). The time evolution of V_{E} was not as simple in the latter case (see Model 9 and 13 in Fig. 3d,e). For this reason, at any time t, we choose $\min(L_0, L(V_E, t))$ as the lithospheric thickness. In our calculation, F_L is defined as

$$F_L = \min(F_{\mathrm{brittle}}, F_{\mathrm{ductile}}), \tag{A5}$$

where F_{brittle} and F_{ductile} are the integrated brittle and ductile strength of lithosphere. F_{brittle} is given as

$$\begin{aligned} F_{\mathrm{brittle}}(\boldsymbol{\omega}) &= \int_0^{L(\boldsymbol{\omega})} \tau_{\mathrm{brittle}}(\boldsymbol{\omega})\, dz \\ &= 0.30 L(\boldsymbol{\omega}) \left[C\left(\varepsilon_{pl}(\boldsymbol{\omega})\right) \cos\phi\left(\varepsilon_{pl}(\boldsymbol{\omega})\right) + P_{\mathrm{mean}} \sin\phi\left(\varepsilon_{pl}(\boldsymbol{\omega})\right) \right], \end{aligned} \tag{A6}$$

where $\boldsymbol{\omega} = (V_{\mathrm{E}}, t)$, $L(\boldsymbol{\omega})$ is the lithospheric thickness, $\min(L_0, L(V_E, t))$, P_{mean} is the mean pressure over $L(\boldsymbol{\omega})$ approximated to the lithostatic pressure at $L(\boldsymbol{\omega})/2$, and C and ϕ are cohesion and internal friction angle that decrease in

magnitude as plastic strain ε_{ps} increases. The factor of 30% in F_{brittle} is the portion of brittle weakening shown in Fig. 1a. ε_{ps} itself is a function of V_{hs} and t and is approximated as

$$\varepsilon_{ps} = 0.1\dot{\varepsilon}$$

$$\varepsilon_{ps} = \sum_{i=1}^{k} \frac{\varepsilon_{ps_{i-1}} + \varepsilon_{ps_i}}{2}(t_i - t_{i-1}) + 1$$

as ε_{ps} with a mean value of 1 due to its initial random distribution between 0.5 to 1.5. The reason for selecting plastic strain rate as 10% of $\dot{\varepsilon}$ is to delay the weakening of F_{brittle} and it did not have major effects on V_{E} trend shown in Fig. 6. F_{ductile} is defined as

$$F_{\text{ductile}} = L(\boldsymbol{\omega})\dot{\varepsilon}\eta_{\text{Lith}}, \tag{A7}$$

where $\dot{\varepsilon}$ is $2V_{\text{E}}/W(\boldsymbol{\omega})$ and η_{lith} is $\exp(Q/RT_{\text{m}})$.

To compute F_{A}, we considered only the regions of large shear strain rates, regions A and B in Fig. A1. We further assumed that the shear flow within each of regions A can be approximated as a Couette flow such that the shear stress is $2\eta_{\text{asth}}V_E/W_{\text{horz}}$ with η_{asth}, the asthenospheric viscosity, being 10^{20} Pa·s. The total contribution to F_{asth} from the two region A's is twice the shear stress times W_{horz}, which is $4\eta_{\text{asth}}V_E$. Similarly, we approximated the vertical inward flow through the bottom boundary in region B (Fig. A1) as Couette flows. If a mirror symmetry is assumed, the right half of the inward flow's contribution to F_{asth} would be $2\eta_{\text{asth}}V_E$. Thus the total contribution from region B is $4\eta_{\text{asth}}V_E$. From the sum of the two components,

$$F_{\text{A}} = 8\,\eta_{asth}\,V_E. \tag{A8}$$

The force balance equation A1 can be rewritten in the following residual form:

$$r_F = F_B - F_L - F_A. \tag{A9}$$

While the equation $r_F(V_E, t) = 0$ can be numerically solved for V_{hs} as a function of t, we instead plot r_F for V_{hs} from 10 to 70 mm/yr and t from 0 to 20 Ma in Figure 6 for intuitive identification of the solution pairs of V_{E} and t from the zero-value contours.

A Python Jupyter notebook for the above calculations and Figure 5 is provided as Supplementary Information.
