# Supplementary material for: Continental rifts losing driving forces can still complete breakup
Source: Sci Rep. 2025 Oct 23;15:37023. doi: 10.1038/s41598-025-19691-3 (PMC12549862; doi:10.1038/s41598-025-19691-3)
Supplement: Supplementary file 2 — Supplementary Information 2. [file 41598_2025_19691_MOESM2_ESM.pdf]

Supplementary information for **Continental rifts losing driving forces can still complete breakup**

Kuruvitage Chameera Chathuranga Silva[1*] and Eunseo Choi[1†]

[1*]Center for Earthquake Research and Information, The University of Memphis, 3890 Central Ave., Memphis, 38152, TN, USA.

*Corresponding author(s). E-mail(s): ksilva2@memphis.edu;
Contributing authors: echoi2@memphis.edu;
†These authors contributed equally to this work.

Supplementary Data

**Parameter**	**Units**	**Upper crust**	**Lower crust**	**Mantle**	
Density (ρ_0)	kg m^{-3}	2700	2900	3300	
Flow law	–	Wet Qtz.	Wet Anth.	Dry Olivine (*diff*, *disl*)	
Visc. Prefactor (A^*)	Pa^{-n}m^{-p}s^{-1}	8.57×10^{-28}	7.13×10^{-18}	2.37×10^{-15}	6.52×10^{-16}
n	–	4	3	1	3.5
Activation energy (Q)	kJ mol^{-1}	223	345	375	530
Activation volume (V)	m^3 mol^{-1}	–	–	10×10^{-6}	18×10^{-6}
Specific heat (C_p)	J kg^{-1} K^{-1}	750	750	750	
Thermal conductivity (k)	W m^{-1} K^{-1}	2.5	2.5	2.5	
Thermal expansivity (α)	K^{-1}	2.5×10^{-5}	2.5×10^{-5}	2.5×10^{-5}	
Heat production (H)	W m^{-3}	10×10^{-7}	2.5×10^{-7}	0	
Grain size (d)	m	–	–	1×10^{-3}	
Grain size exponent (p)	–	–	–	3	–
Cohesion (C)	Pa	$80^{\dagger}$ $20^{\dagger}$ $80^{\dagger}$	$80^{\dagger}$ $20^{\dagger}$ $80^{\dagger}$	$80^{\ddagger}$ $20^{\ddagger}$	$80^{\ddagger}$ $20^{\ddagger}$
Angle of friction (ϕ)	°	30	30	30	

Supplementary Table 1: Material densities, thermal, and viscous flow law parameters. † Indicates crustal properties divided into three layers. ‡ Indicates mantle properties divided into four layers.

Supplementary Movies 1-11
Description: Movies 1-11: Time-series animations of CTM, Model 22,5,6,9,10,11,12,13,20, and 21 showing the progression to continental breakup or rift failure. Each frame displays the evolving geotherm (temperature contours), and viscosity evolution.

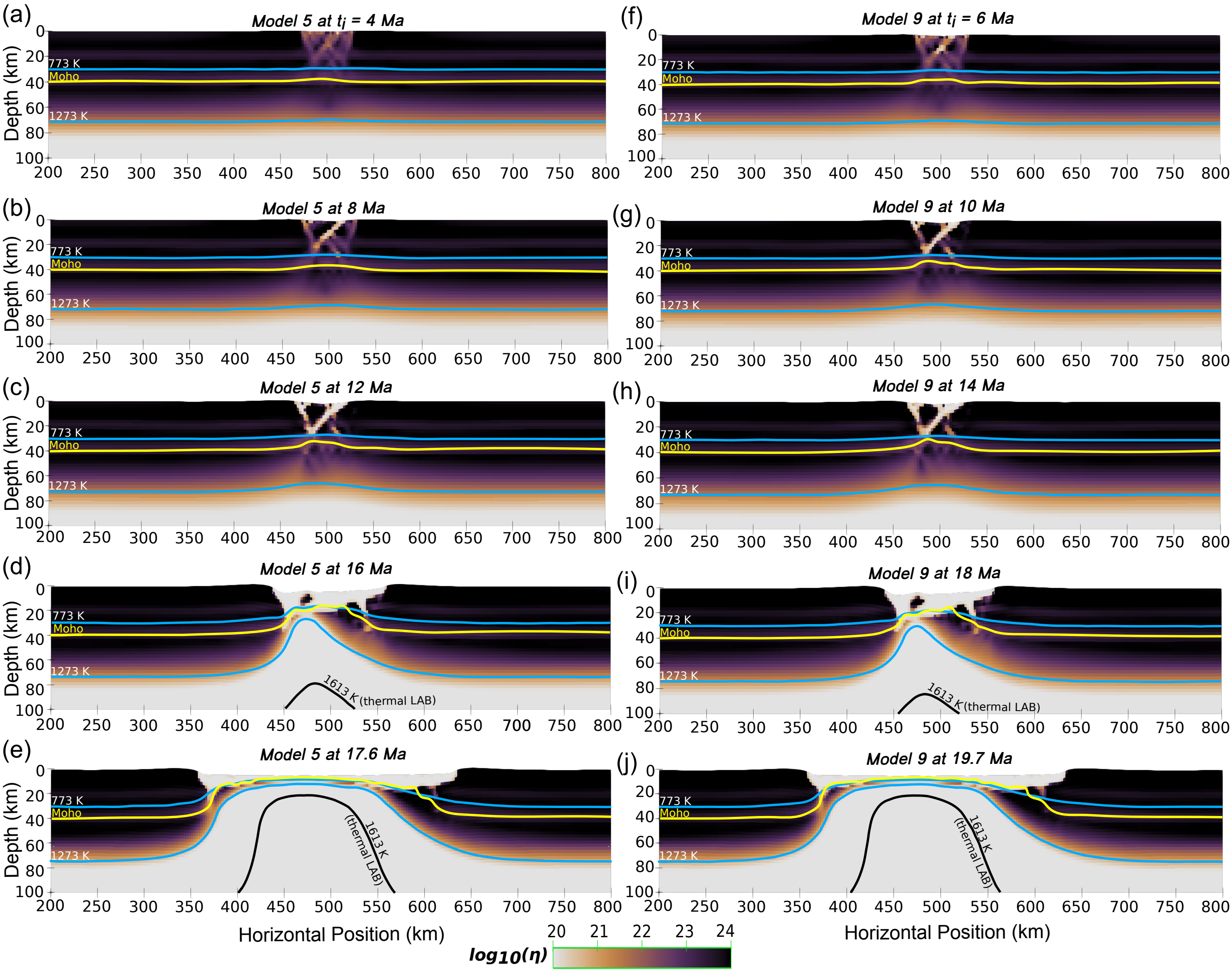

Supplementary Figure 1: (a-e) Viscosity evolution of Model 5 at 4,8,12,16, and 17.6 Ma with isotherms at 773, 1273, and 1613 K. The 1613 K isotherm is identified with the thermal lithosphere-asthenosphere boundary. The Moho is represented by solid yellow line. Also shown is accumulated plastic strain distribution essentially demarcating the broadened weak zone. (f-j) Same as (a-e) but for Model 9 at 6,10,14,18, and 19.7 Ma.

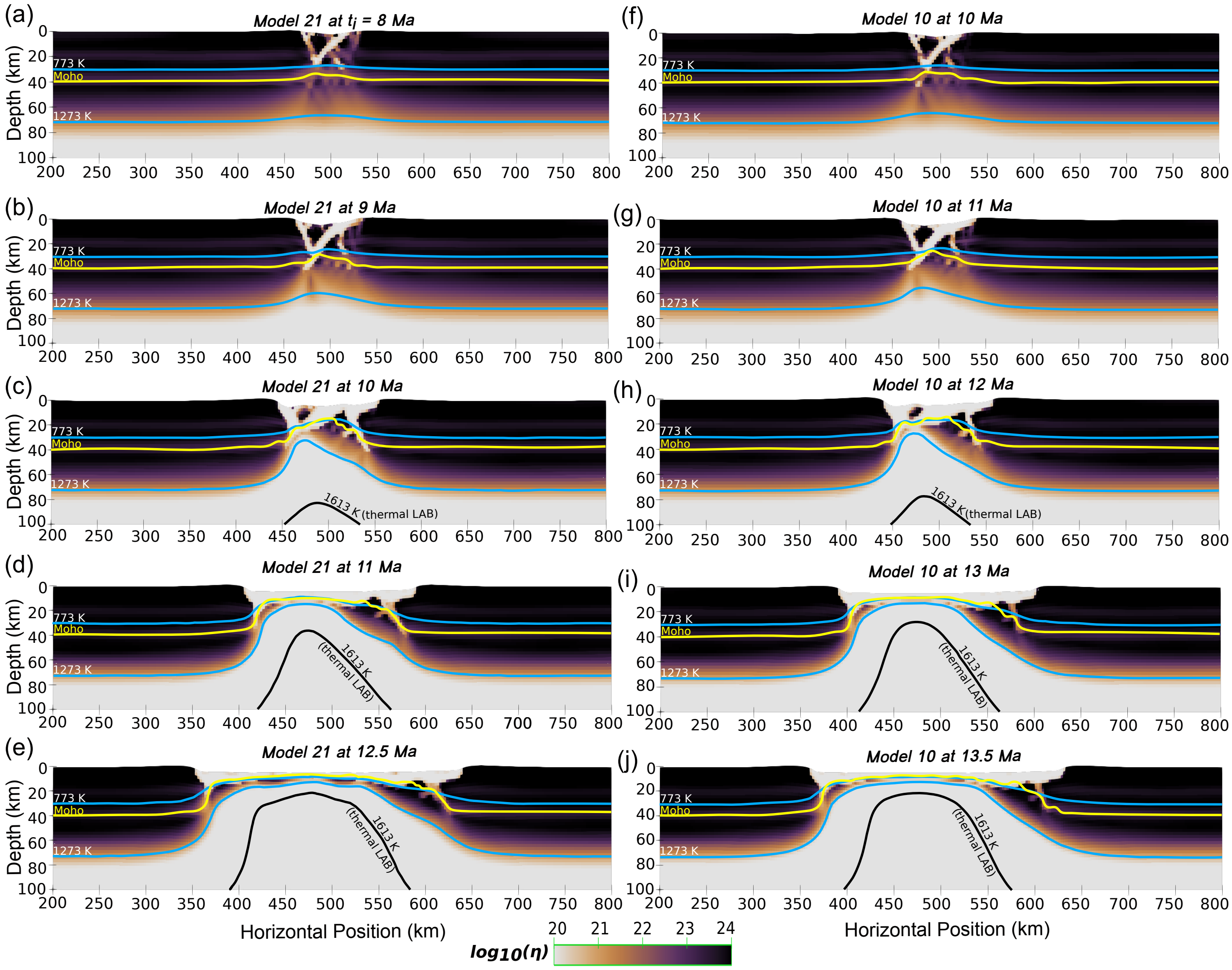

Supplementary Figure 2: (a-e) Viscosity evolution of Model 21 at 8,9,10,11, and 12.5 Ma with isotherms at 773, 1273, and 1613 K. The 1613 K isotherm is identified with the thermal lithosphere-asthenosphere boundary. The Moho is represented by solid yellow line. Also shown is accumulated plastic strain distribution essentially demarcating the broadened weak zone. (h-j) Same as (a-e) but for Model 10 at 10,11,12,13, and 13.5 Ma.

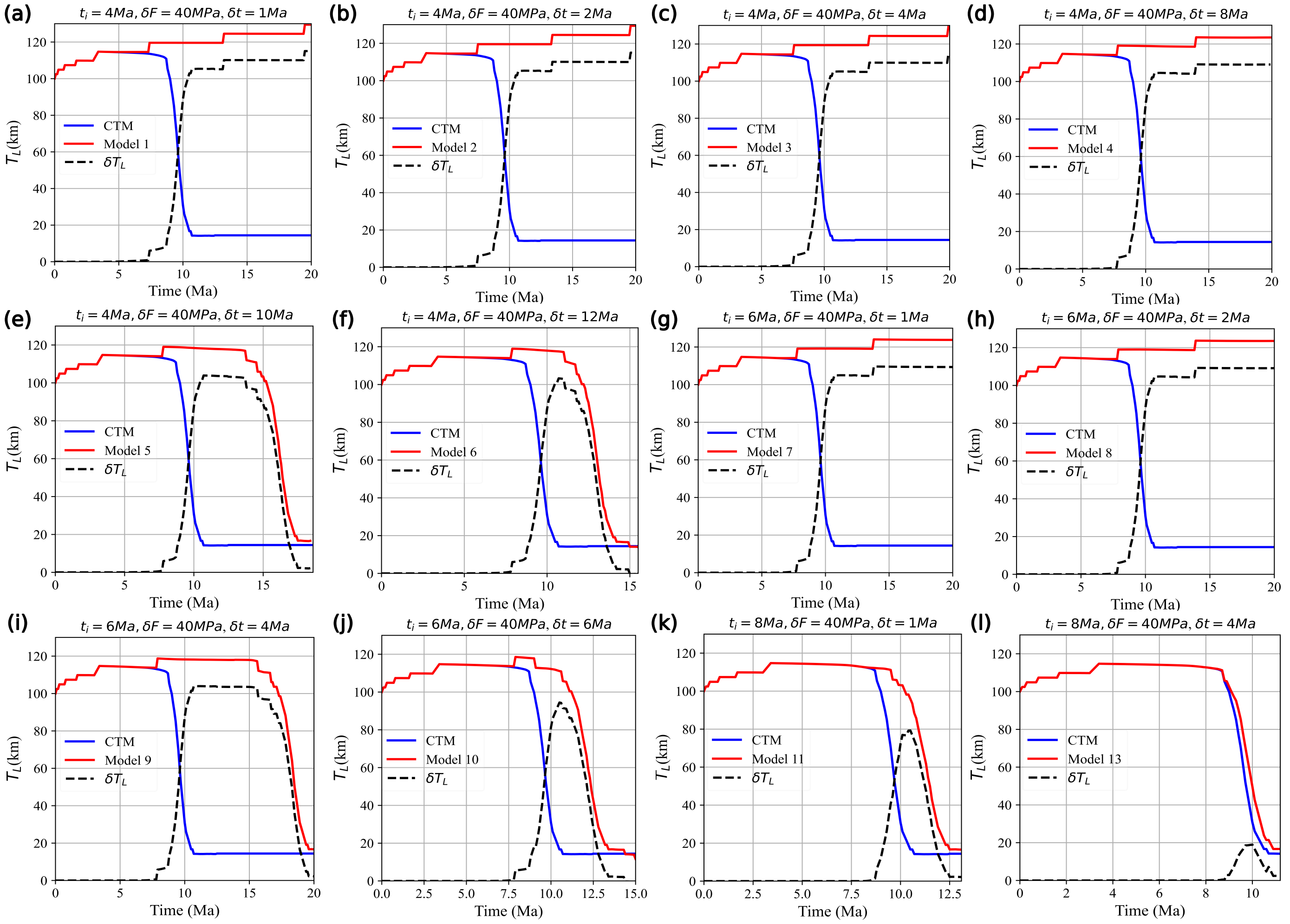

Supplementary Figure 3: Lithospheric thickness evolution in time-dependent traction models with $\delta F = 40$ MPa, compared against the constant force model (CFM). δT_L denotes the change in lithospheric thickness relative to the CFM. Panels (a–f) show models with a traction reduction duration of $\delta t_i = 4$ Ma, (g–j) correspond to $\delta t_i = 6$ Ma, and (k–l) represent $\delta t_i = 8$ Ma.

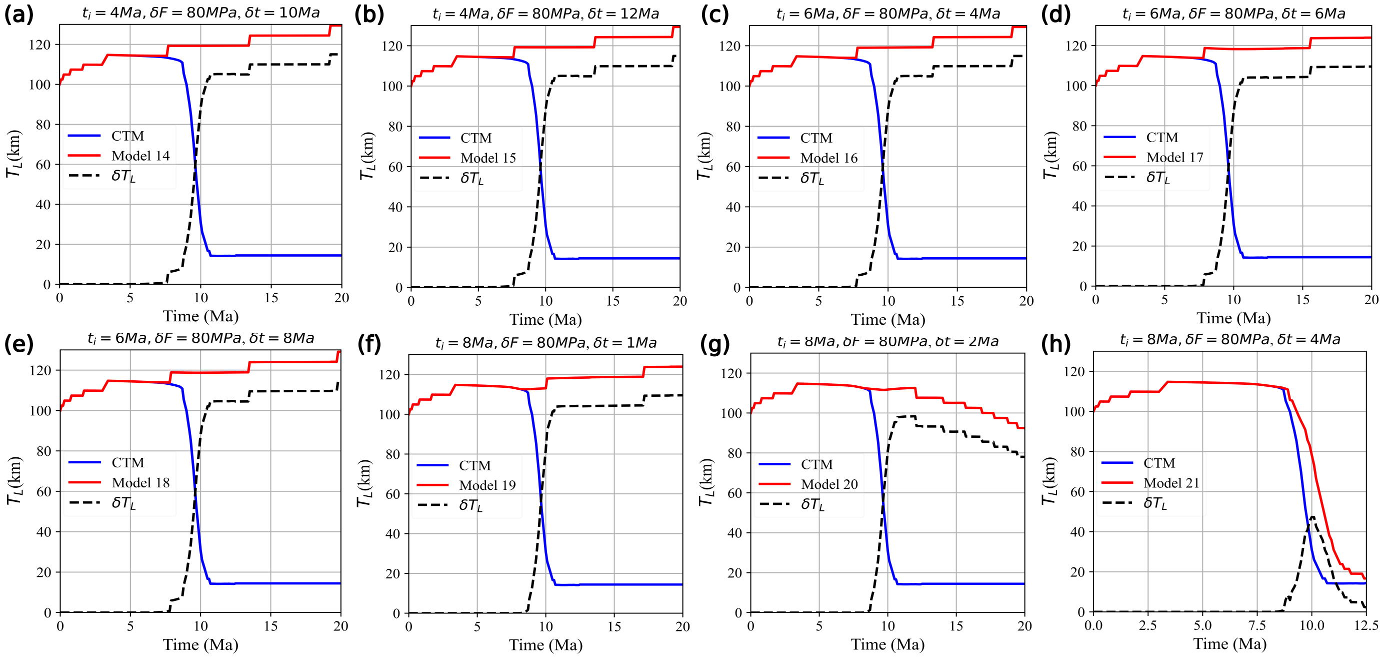

Supplementary Figure 4: Lithospheric thickness evolution in time-dependent traction models with $\delta F = 80$ MPa, compared against the constant force model (CFM). δT_L denotes the change in lithospheric thickness relative to the CFM. Panels (a–b) show models with a traction reduction duration of $\delta t_i = 4$ Ma, (c–e) correspond to $\delta t_i = 6$ Ma, and (f–h) represent $\delta t_i = 8$ Ma.

Continental geotherm

we first present in detail how the initial geotherm is calculated in ASPECT[1, 2]. ASPECT uses the solution to the stead-state heat equation with a depth-varying distribution of volumetric heat source. In practice, ASPECT adopts the analytic solution presented in Chapman's 1986 paper [3], the one simplified for a layered structure with constant conductivity and volumetric heat generation in each layer. The depth distribution of temperature ($T(z)$) is given as

$$T(z) = T_T + \frac{q_T}{k} z - \frac{Az^2}{2k} \tag{1}$$

where $T(z)$ is the temperature distribution over depth, z, T_T and q_T are the temperature and heat flux at the layer surface, k is thermal conductivity, and A is volumetric radiogenic heat production. For a layer thickness Δz, the basal temperature (T_B) and heat flux (q_B) are given as

$$\begin{aligned} T_B &= T_T + \frac{q_T}{k}\Delta z - \frac{A\Delta z^2}{2k}, \\ q_B &= q_T - A\Delta z. \end{aligned} \tag{2}$$

T_B and q_B are then used for the next layer. This process is repeated successively for all the layers considered. In our study, we used T_T of 273 K and q_T of 55 mW/m^2 for the upper crust layer, successively computing the lower crust and mantle geotherm. The thermal conductivity and radiogenic heat production assumed for each layer are listed in Supplementary Table S1.

References

[1] Heister, T., Dannberg, J., Gassmöller, R. & Bangerth, W. High accuracy mantle convection simulation through modern numerical methods – II: realistic models and problems. *Geophysical Journal International* **210**, 833–851 (2017).

[2] Kronbichler, M., Heister, T. & Bangerth, W. High accuracy mantle convection simulation through modern numerical methods: High accuracy mantle convection simulation. *Geophysical Journal International* **191**, 12–29 (2012).

[3] Chapman, D. S. Thermal gradients in the continental crust. *Geological Society, London, Special Publications* **24**, 63–70 (1986). URL https://www.lyellcollection.org/doi/10.1144/GSL.SP.1986.024.01.07.
